# Damage Monitoring of Engineered Cementitious Composite Beams Reinforced with Hybrid Bars Using Piezoceramic-Based Smart Aggregates

**DOI:** 10.3390/s22197184

**Published:** 2022-09-22

**Authors:** Hui Qian, Yuqing Zhang, Yuechang Li, Jundong Gao, Jianxue Song

**Affiliations:** School of Civil Engineering, Zhengzhou University, Zhengzhou 450001, China

**Keywords:** PZT, smart aggregate, ECC beam, damage monitoring

## Abstract

In order to explore the crack development mechanism and damage self-repairing capacity of ECC beams reinforced with hybrid bars, the smart aggregate-based active sensing approach were herein adopted to conduct damage monitoring of ECC beams under cyclic loading. A total of six beams, including five engineered cementitious composite (ECC) beams reinforced with different bars and one reinforcement concrete counterpart, were fabricated and tested under cyclic loading. The ultimate failure modes and hysteresis curves were obtained and discussed herein, demonstrating the multiple crack behavior and excellent ductility of ECC material. The damage of the tested beams was monitored by smart aggregate-based (SA) active sensing method, in which two SAs pasted on both beam ends were used as actuator and sensor, respectively. The time domain analysis, wavelet packet-based energy analysis and wavelet packet-based damage index analysis were performed to quantitatively evaluate the crack development. To evaluate the self-repairing capacity of the beams, a self-repairing index defined by the difference of damage index at loading and unloading peak points was proposed. The results in time domain and wavelet packed analysis were in close agreement with the observed crack development, revealing the feasibility of smart aggregate-based active sensing approach in damage detection for ECC beams. Especially, the proposed damage self-repairing index can describe the same structural re-centering phenomena with the test results, showing the proposed index can be used to evaluate the damage self-repairing capacity.

## 1. Introduction

Engineered cementitious composites (ECCs) are fiber-reinforced cementitious composite materials [1,2], where an appropriate amount of polyvinyl alcohol (PVA) fibers are randomly distributed to form a three-dimensional space supporting system. Hence, the tensile strain capacity, the toughness, the durability and the impact fatigue of concrete members are significantly enhanced [3,4,5]. Specifically, two of the most important mechanical characteristics of ECC are the quasi-strain hardening properties and the multiple micro-cracking behavior with self-controlled crack widths [3,6]. The ultimate tensile strain attained by ECC is 200–600 times greater than that of regular concrete, and the multiple cracking behavior of ECC is distinguished from that of regular concrete. However, the damage monitoring of ECC is not well studied in the literature despite its great importance.

There have been several techniques for damage monitoring or health monitoring of structures in the past few decades [7]. Piezoceramic-based smart aggregates are multi-functional and can perform various tasks [8]. The PZT (Lead Zirconate Titanate) is the most popular piezoceramic material due to its strong piezoelectric effect [9], high bandwidth [10,11], fast response [12,13], and availability in different forms [14,15]. The applicability of PZT smart aggregates to health monitoring and damage detection of various civil structures has been demonstrated by experimental results. Gu et al. [16] conducted the early-age strength monitoring of concrete cylinder specimens and predicted the concrete strength development based on the output voltage of the sensors embedded into the concrete structures. Jiang et al. [17] presented a stress wave-based active sensing method to detect the crack in FRP-reinforced concrete beams, and the results show that the developed piezoceramic-based active sensing method can monitor the crack-induced damage and estimate the process of damage degree in real-time. Song et al. [18] developed a smart aggregate-based impact detection and evaluation system, which had been used to detect the impacts on a concrete beam. Song et al. [19] also performed the structural health monitoring of a specially designed concrete bent-cap, indicating that the smart aggregate is able to capture the moment of concrete cracking. More recently, smart aggregates have been increasing employed in the monitoring field, involving the strength monitoring of early age concrete [20], impact detection and evaluation [21], health monitoring [22,23,24,25,26,27] and damage detection [28,29].

On the other hand, PZT smart aggregates were experimentally validated to be applicable to other fields, such as bolt looseness monitoring [30,31,32,33], soil freeze–thaw process monitoring [34], monitoring of water content in sandy soil [35,36], soil compaction monitoring [37], degree of water permeability [38,39], and damage diagnosis of hydraulic structure [40]. At the same time, to meet the monitoring environment under different conditions, the forms of piezoelectric intelligent aggregates are becoming more and more diverse. Gao et al. [41] designed, fabricated, and tested a novel embeddable tubular smart aggregate (TSA) based on a piezoceramic tube for use in two dimensional (2D) structures, and through test results showed that the proposed TSA is suitable for monitoring the health condition of a 2D concrete structure. Lu et al. [42] developed a novel piezoceramic stack-based smart aggregate (PiSSA) with piezoceramic wafers in series or parallel connection to increase the efficiency and output performance over the conventional smart aggregate. Moreover, the research study on wireless smart aggregates (WSAs) was conducted by Yan et al. [43], and the efficiency of the WSA health monitoring system was experimentally validated in a bridge health monitoring system [44]. Voutetaki et al. [45] detected and evaluated the damage severity of shear critical concrete beams with a new portable real-time wireless impedance monitoring system.

In order to explore the crack development in ECC beams, the smart aggregate-based active sensing approach are herein adopted to conduct damage monitoring of ECC beams under cyclic loading. Five beams made of ECC materials reinforced with different types of hybrid reinforcement materials and one reinforcement concrete counterpart were designed and fabricated. The one-way cyclic loading test results of six beams are presented to discuss the reinforcement effect of composited materials on crack development, together with the self-centering effect. In addition, the crack development and self-centering effect are investigated by the time domain analysis and wavelet packet analysis.

## 2. Principle of Damage Monitoring

### 2.1. Smart Aggregate-Based Active Sensing Approach

**Figure 1:**
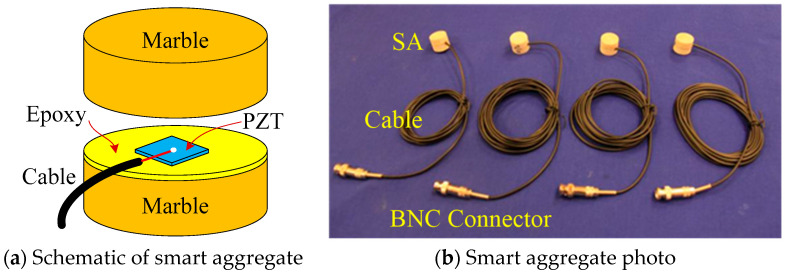
Piezoceramic-based smart aggregate.

In general, piezoceramic materials cannot be directly used in structural health monitoring, owing to the inherent fragility. A smart aggregate is thus designed by sandwiching the PZT patch into two marble blocks with epoxy, as illustrated in Figure 1a. Meanwhile, the cable with a Bayonet Neill–Concelman (BNC) connector is soldered to the PZT patch of the smart aggregate, as shown in Figure 1b. The smart aggregates are employed in the active sensing approach. Specifically, one smart aggregate connected to the waveform generator is used as an actuator to send excitation waves, and other distributed smart aggregates are regarded as sensors to simultaneously detect the propagated signals [34]. The values of wave amplitude and transmission energy will decrease with the occurrence of the cracks or damage inside the concrete, and the dropped values are associated with the degree of damage inside.

In this paper, the damage development of ECC beams under cyclic loading is monitored by the active sensing approach, wherein the cracks or damage inside the tested beams can be reflected by the signals recorded by the sensors. During the damage monitoring of the ECC beam, the stress wave will change with the generation and closure of cracks. The signal received by the collector can be analyzed by the wavelet packet algorithm in terms of energy and damage index.

### 2.2. Wavelet Packet Analysis

**Figure 2:**
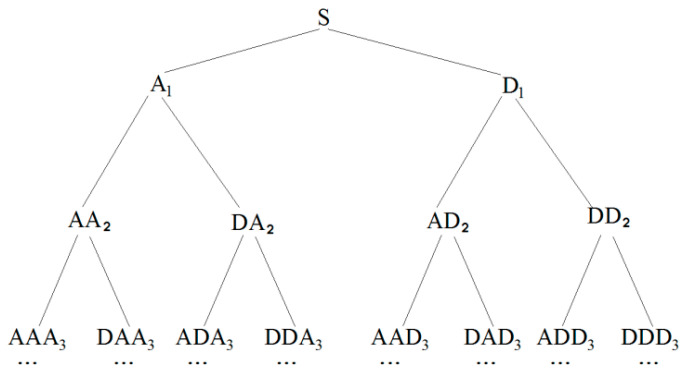
Wavelet packet decomposition signal.

The principle of wavelet packet analysis can be explained by the Figure 2, wherein the sensor signal S is decomposed by an *n*-level wavelet packet decomposition into 2*^n^* signal sets {*X*_1_, *X*_2_,…, X_2_*^n^*}. *X_j_* is given by

(1) $$X_{j}=[x_{j,1}^{}+x_{j,2}^{}+\ldots +x_{j,m}^{}]$$

where *m* is the number of sampling data and *j* is the frequency band (*j* = 1, …, 2*^n^*). The energy (*E_i_*_, *j*_) of the band signal *j* at time *i* is defined as,

(2) $$E_{i,j}={\left\|X_{j}\right\|}_{2}^{2}=x_{j,1}^{2}+x_{j,2}^{2}+\ldots +x_{j,m}^{2}(j=1,\;2,\;3,\;\ldots ,2^{n})$$

where *i* is the time index.

The difference between the signatures of health and damaged states have been commonly compared by root mean-square deviation (RMSD), which is the widely used damage index for health monitoring of concrete structures. The energy vector at health states *E*_0,*j*_ = [*E*_0,1_, *E*_0,2_,…, *E*_0,2_*^n^*], whilst the energy vector for the damaged data at time index *i* is marked as,

(3) $$E_{i}=\left[E_{i,1},E_{i,2},\ldots E_{i,2^{n}}\right]$$

Therefore, the damage index at time *i* can be defined as,

(4) $$D=\sqrt{\frac{{\sum_{j=1}^{2^{n}}\left(E_{i,j}-E_{0,j}\right)}^{2}}{\sum_{j=1}^{2^{n}}E_{0,j}^{2}}}$$

The transmission energy loss portion caused by structural damage can be quantitatively evaluated by the damage index *D*. When the concrete structure stays in a healthy state, the damage index is 0. While the damage index is larger than the initial value, indicating that damage appears in the concrete structures. Greater index indicates more serious damage. The value of damage index at the complete failure of a concrete structure is close to 1.

### 2.3. Damage Self-Repairing Index

For the re-centering structures, in order to evaluate the damage self-repairing capacity of the structure member, a damage self-repairing index is proposed in this paper. The self-repairing effect of structural members can be evaluated by proposed index, which is defined by the ratio of the difference of damage index at loading peak point, and unloading and the damage index at loading peak point. Therefore, the damage self-repairing index can be defined as,

(5) $$R_{m}=\frac{D_{m,l}-D_{m,n}}{D_{m,l}}$$

where $R_{m}$ represents the self-repairing ratio of damage during the cyclic loading; *m* stands for a certain cycle of structure loading; *l* stands for the loading peak point during the cycle; and *u* stands for the unloading phase during the cycle. If *R_m_* =1, it indicates the structure has a good self-repairing effect. While if *R_m_* =0, it indicates that the structure has no self-repairing effect.

By comparing the damage self-repairing index of each loading cycle, whether the structure has self-repairing effect can be obtained. By comparing the damage self-repairing index of different components with the same loading cycle, we can obtain the self-repairing condition between different components.

## 3. Test of ECC Beams

### 3.1. Test Specimens

**Figure 3:**
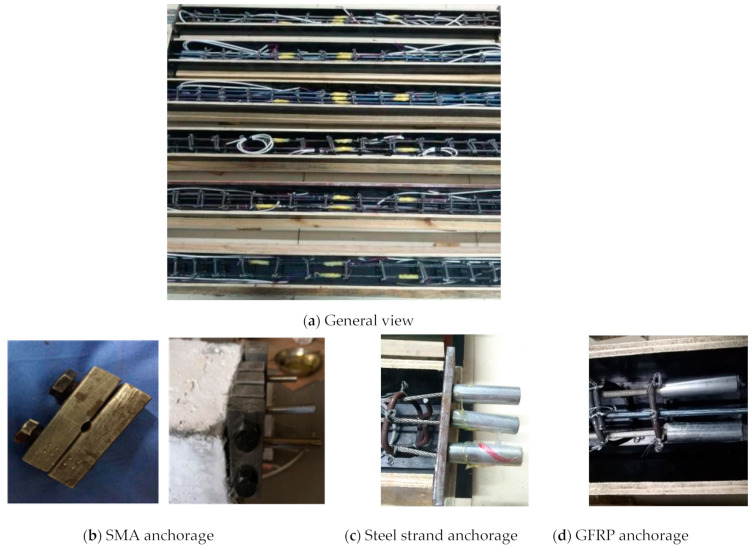
Photo of six beam specimens.

**Figure 4:**
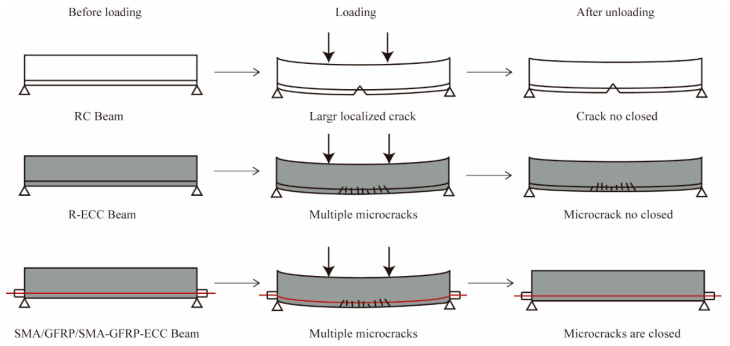
Schematic drawing of crack changes.

A total of six concrete beams were designed with the same cross-sectional area of 100 mm × 100 mm, and the length was set to 1100 mm, as shown in Table 1. The specimen RC is a reinforced concrete beam, while another five specimens are made of ECC material, and are reinforced with steel rebars, steel strands, glass fiber reinforced plastics (GFRP) rods, shape memory alloy (SMA) rods, and both GFRP and SMA rods, respectively, as shown in Figure 3. The material of both reinforcements and stirrups is HRB 400, and the arrangements of stirrups are the same for six specimens. The crack changes of six beams can be schematically illustrated by Figure 4.

### 3.2. Materials

The compressive strength of concrete was measured by the uniaxial compression test of three cubes with the same size of 150 mm × 150 mm × 150 mm, and the average value is equal to 44.02 MPa. The ECC material employed in the specimens was mixed with 2% of polyvinyl alcohol (PVA) fiber. The values of both tensile strength and compressive strength were determined, as summarized in Table 2.

**Figure 5:**
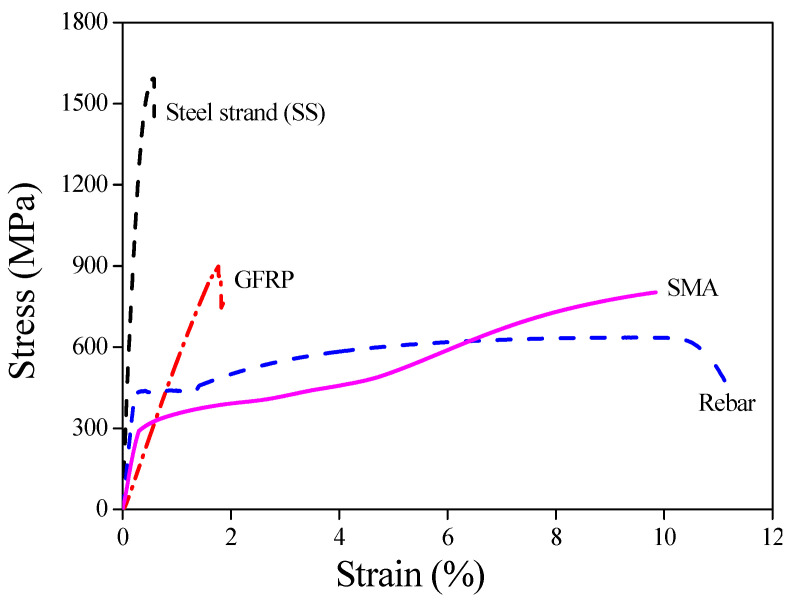
Stress–strain relationship of four different materials under uniaxial tensile loading.

The SMA material selected in this study is a Ni-Ti alloy (56.35% Ni) with a diameter of 8 mm, and the diameter of GFRP and steel rebar is also 8 mm, while the nominal diameter of steel strand is 4.5 mm. The uniaxial tensile test at room temperature was performed to four kinds of reinforcement material, and the stress–strain relationships are presented in Figure 5. The brittle failure occurs for both steel strand and GFRP when the values of the tensile strength reach 1592.15 MPa and 881.35 MPa, respectively. The steel rebar and SMA experience four stages, involving the elastic stage, the elastoplastic stage, the plastic stage and the failure stage, the maximum tensile strengths of which reach 635.31 MPa and 802.35 MPa, respectively.

### 3.3. Test Setup and Loading Procedure

**Figure 6:**
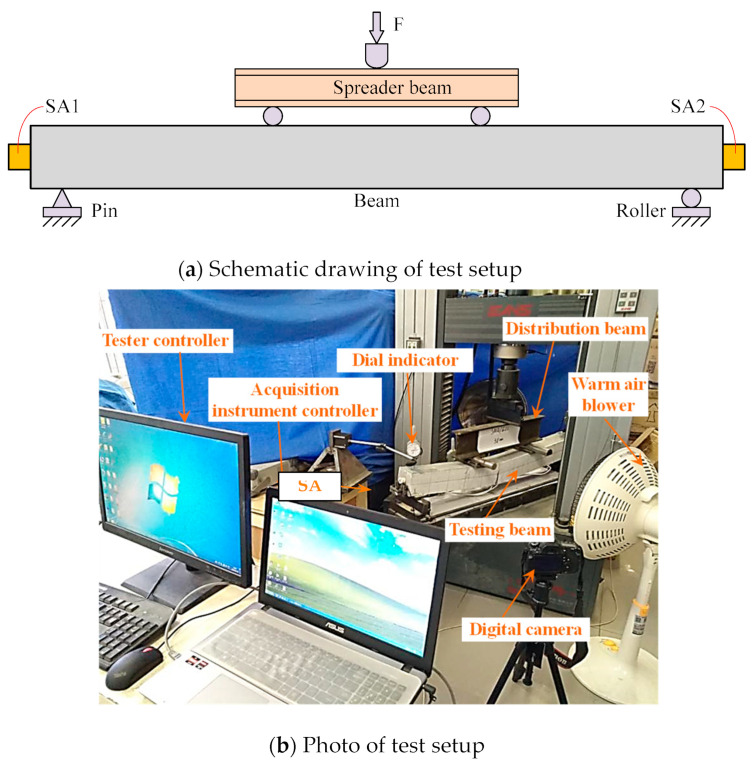
Test setup.

The setup for the cyclic loading tests on six beam specimens is displayed in Figure 6a, wherein the span was designed as 1000 mm. Each beam specimen was tested under four-point bending condition. The load applied at mid-span was transmitted to the beam specimen by the spreader beam, and both beam ends were simply placed at two steel bearings including the pin support and the roller support. Two smart aggregates marked as SA1 and SA2, were attached to both ends of the beam with epoxy to detect the damage development and self-centering effect, wherein SA2 was responsible for recording the wave signal excited by SA1. Figure 6b is a picture of test setup.

**Figure 7:**
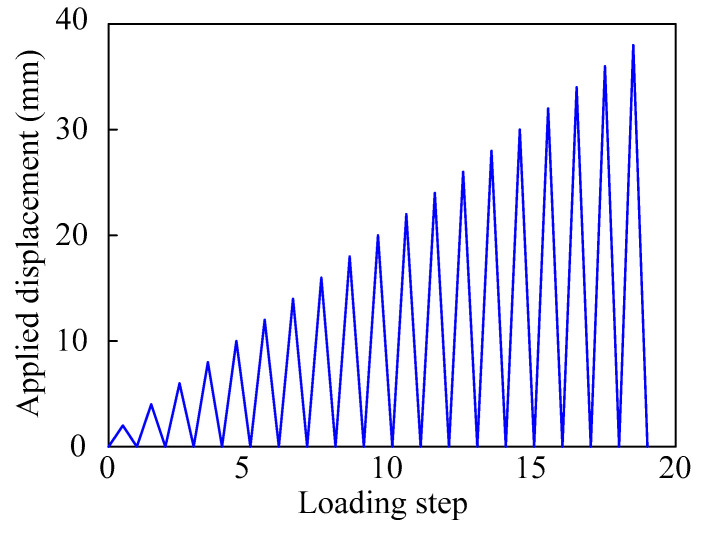
Loading protocol.

All the specimens were loaded according to the same loading protocol, as displayed in Figure 7. Displacement control was employed, and the displacement applied at mid-span gradually increased in equal increment of 2 mm. Only one cycle at each step was required. The loading process was terminated until the load decreased to 85% of the peak load.

**Figure 8:**
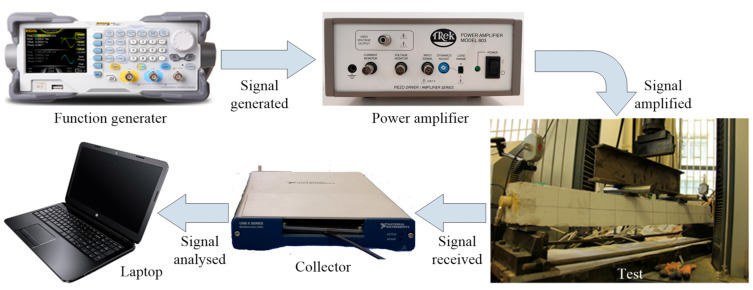
Schematic of test system for damage monitoring.

The employed test system for damage monitoring is schematically shown in Figure 8. During the loading procedure, the repeated swept sine wave at a frequency range of 100 Hz to 130 kHz was generated by the function generator and then amplified by power amplifier, and the PZT smart aggregate SA1 was stimulated. Afterwards, the stress wave propagated through the beam and was received by the sensor SA2. The detected wave signals were recorded and analyzed by the collector and the laptop with supporting software, respectively. It should be mentioned that the initial healthy state of each specimen monitored before applying load was supposed as the benchmark, and the subsequent damage development monitoring for the beam under cyclic loading was carried out. At the end of loading and unloading for each cycle, the signal propagated through the specimen was recorded by the damage monitoring system.

## 4. Experiment Results and Analysis

### 4.1. Experiment Results

**Figure 9:**
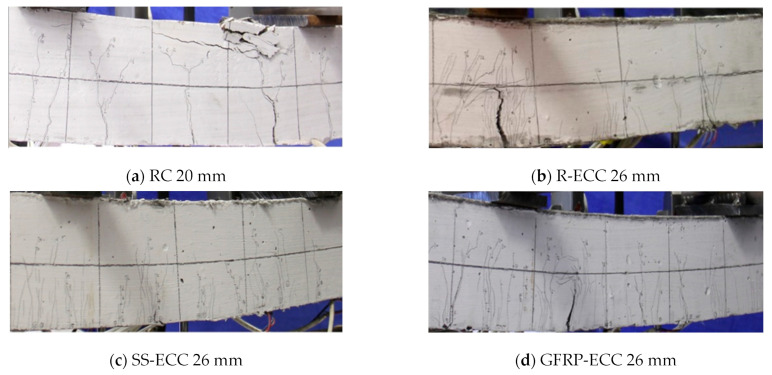

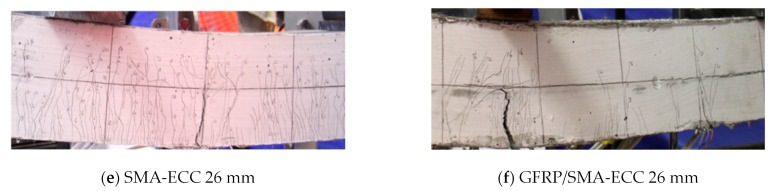
Failure modes and the crack widths of tested beams.

The obtained failure modes and the crack widths of six tested beams are illustrated in Figure 9. Noticeable cracks from the tension zone at beam bottom until the neutral axis can be observed for each specimen. The termination of the loading process is associated with the occurrence of a major crack, except for specimen SS-ECC. This can be explained by the fact that the rupture of pretensioned steel strands occurred for specimen SS-ECC. It was also noted that only the concrete in the compression zone of the specimen RC was crushed, another five beam specimens exhibit multiple cracking behavior.

**Figure 10:**
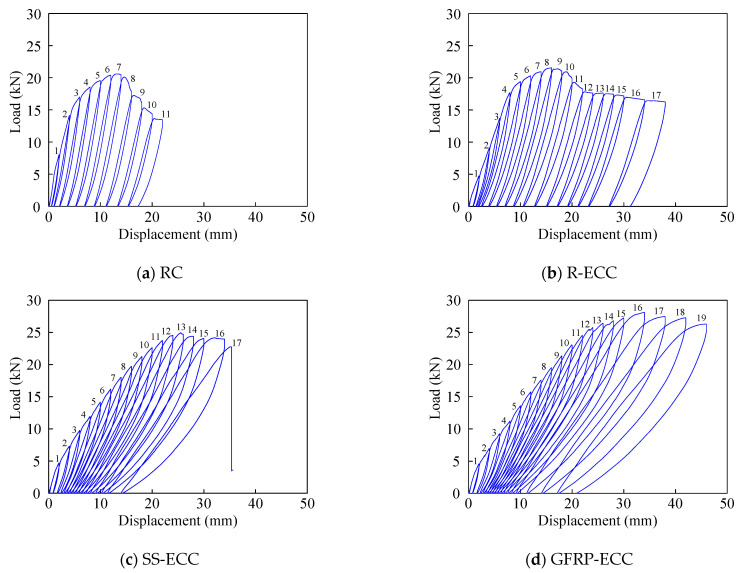

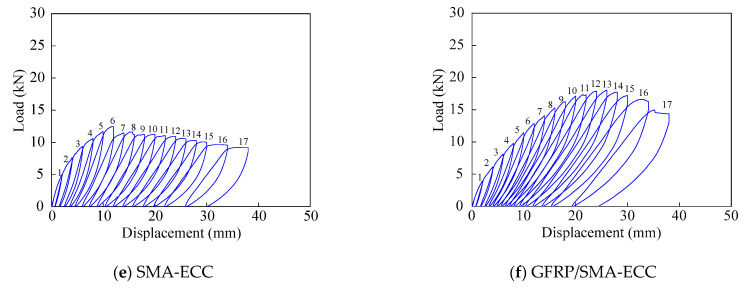
Hysteresis curves of tested beams.

Hysteresis curves of six specimens are illustrated in Figure 10, wherein the vertical coordinate is the applied load, and the abscissa is the displacement at the mid-span of the spreader beam. It is clear that larger displacement amplitude can be achieved by the beam specimens with ECC material, compared with the concrete beam specimen RC. Specimen GFRP-ECC experiences the largest deformation, and the applied load is also maximum among the tested beams, indicating the excellent load-carrying capacity. The load applied to specimen SS-ECC drops sharply owing to the fracture of steel strands. It should be mentioned that the load of beam specimen SMA-ECC is the least.

**Figure 11:**
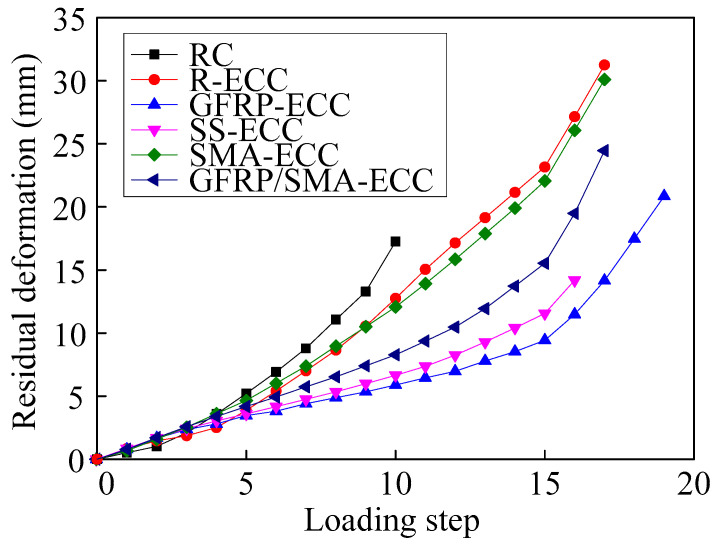
Residual deformation with increasing loading step.

The residual deformation of the beam with increasing loading step is plotted in Figure 11. Significant residual deformation of six beams can be observed, implying the occurrence of a poor self-centering effect. At the same loading level, the residual deformation of the reinforced concrete beam specimen RC is higher than the others, while which of specimen GFRP-ECC is the least. It is noted that the values of specimens R-ECC and SMA-ECC in terms of residual deformation are the close to each other, and the cumulative residual deformation to these two beams are the most significant.

### 4.2. Damage Monitoring Results

**Figure 12:**
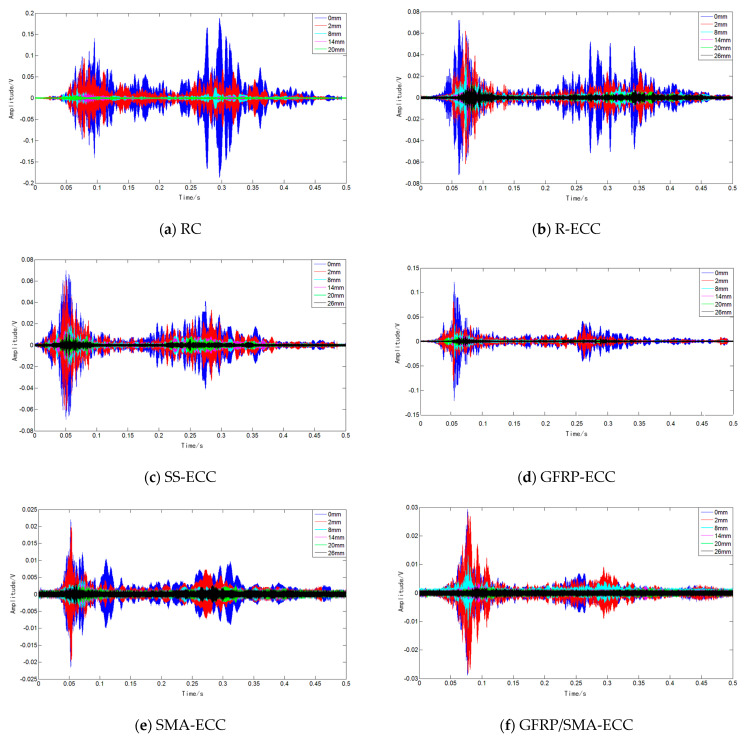
Time-domain analysis.

Time-domain analysis is employed herein to reflect the development of cracks. The received sensor voltage signals of six beam specimens are shown in Figure 12, where the signal amplitudes collected at specified displacement level are presented. It can be clearly found that the signal strength in the case of no crack is greater than that at damage status. Furthermore, the amplitudes of sensor voltage decrease with increasing displacement, and the decline amount is gradually narrowing, reflecting that cracks develop significantly until the failure of the beam.

**Figure 13:**
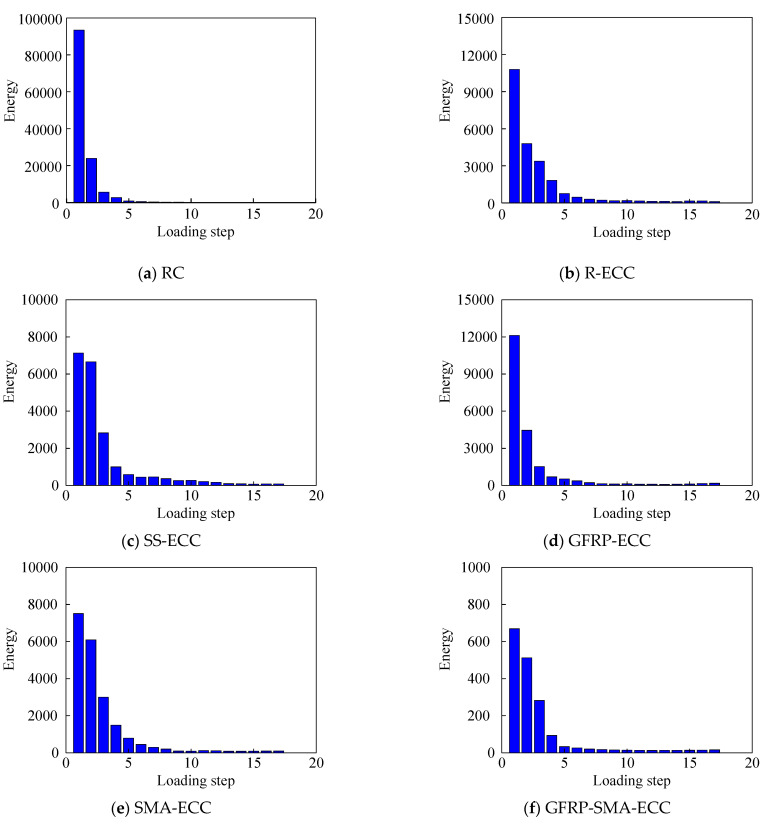
Energy indices for the six beams.

Figure 13 displays the energy indices with increasing loading cycle. It is clear that the energy propagated through the beam length gradually decreases with the development of cracks. The detected energy reduced to 25.6% of that at the health status, when the displacement applied to beam RC is equal to 2 mm, indicating the occurrence of crack, as illustrated in Figure 13a, wherein the final remaining energy is less than 1% of that in the initial state. Compared with detected energy at the initial status, the values of observed energy loss portion for five beam specimens with ECC material after the first loading cycle are equal to 56%, 6.6%, 63.3%, 18.9% and 23.4%, respectively, and the final energy ratios range from 1% to 10%. Therefore, it can be found that the energy loss of specimen R-ECC is less serious than its concrete counterpart RC, revealing that the development of cracks can be effectively suppressed by ECC materials. With the completion of the first loading step, the energy loss portion of beam SS-ECC among five ECC beams is the least, indicating the good reinforcement effect of steel strands. On the other hand, a poor reinforcement effect of GFRP is observed, since the beam GFRP-ECC displays the most apparent energy loss.

**Figure 14:**
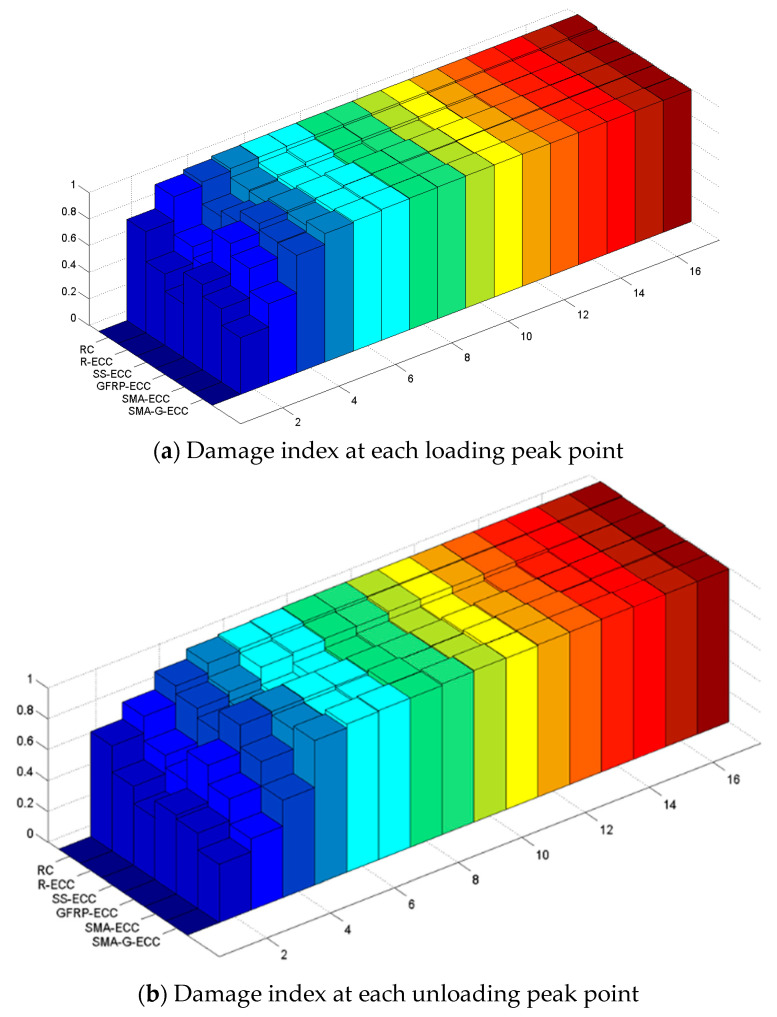
Damage index.

The values of damage indices calculated by wavelet packet analysis are shown in Figure 14, including the values corresponding to both loading and unloading peak points. The initial value of damage index is equal to zero since each beam specimen is in health status. The increment of the damage index correlates with the increment of the loading step. Specifically, the damage index increases greatly with the application of the first-level loading, and the increment of beam RC is largest among all specimens. After the fifth-level loading, the values of damage index for six tested beams are very close to one, indicating extreme structural damage.

**Figure 15:**
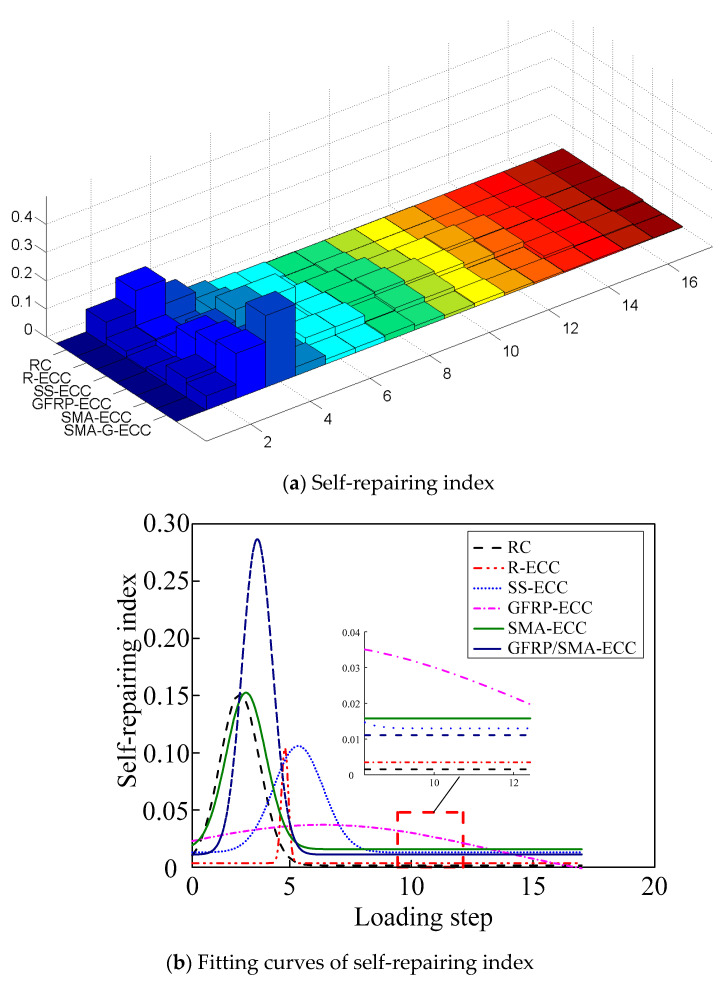
Self-repairing index.

The development of self-repairing index is herein shown in Figure 15, including both histogram and fitting curves. It is evident that the beam GFRP-ECC experiences mild self-repairing owing to the small values of self-repairing index throughout the loading process. Meanwhile, another five specimens exhibit noticeable self-repairing effect in the initial five levels of cyclic loading, and then drops sharply to near zero, indicating that no self-repairing phenomenon can be observed in the later stage. The unexpected self-repairing phenomenon may be explained by the fact that the unexpected adhesion effect between ECC concrete and hybrid bars was noticed owing to the smooth surfaces of hybrid bars. In addition, tested beam specimens without enough reinforcements yielded in the initial five loading levels, and experienced plastic deformation with growing loading cycles. Hence, the residual displacement was remarkable in the later loading stage, and little self-repairing phenomenon was observed.

## 5. Conclusions

The smart aggregate-based active sensing approach is employed to monitor the crack development of six concrete beams under cyclic loading. The results of failure modes, hysteresis cures and residual deformation were analyzed in detail. The time domain analysis and wavelet packet analysis in terms of energy indices and damage index were conducted, and the self-centering effect of tested beams were evaluated. The following conclusions can be drawn:

(1) Noticeable multiple crack behavior and better ductility was observed in beams with ECC. The ECC beam strengthened with GFRP exhibited favorable performance in terms of the load-carrying capacity, ductility, residual deformation and self-centering effect.

(2) The damage monitoring results were consistent with the observed crack development, indicating the feasibility of damage detection for ECC beams using smart aggregate-based active sensing approach. The experimental results provided the basis for the application of PZT smart aggregates in the ECC structures.

(3) The proposed damage self-repairing index can describe the same structural re-centering phenomenon with the test results, showing the proposed index can be used to evaluate the damage self-repairing capacity.

## Figures and Tables

**Table 1:** Specimens’ geometric figures.

Specimen	Cross-Section (mm × mm)	Length (mm)	Reinforcement Srrangement (mm)	Stirrups (mm)
RC	100 × 100	1100	2*φ*8 Steel Bars	*φ*6@80/100
R-ECC	100 × 100	1100	2*φ*8 Steel Bars	*φ*6@80/100
SS-ECC	100 × 100	1100	3*φ*4.5 Steel Strands	*φ*6@80/100
GFRP-ECC	100 × 100	1100	2*φ*8 GFRP Rods	*φ*6@80/100
SMA-ECC	100 × 100	1100	2*φ*8 SMA Rods	*φ*6@80/100
GFRP/SMA-ECC	100 × 100	1100	2*φ*6 GFRP Rods + 1*φ*8 SMA Rods	*φ*6@80/100

**Table 2:** Material properties of ECC.

Compressive Strength (MPa)	Tensile Cracking Strength (MPa)	Tensile Cracking Strain (%)	Ultimate Tensile Strength (MPa)	Ultimate Tensile Strain (%)
26.86	2.17	0.067	4.30	2.89

## Data Availability

The data presented in this study are available on request from the corresponding author.
